# Multifunctional Magnetic Nanowires and Nanotubes

**DOI:** 10.3390/nano12081308

**Published:** 2022-04-11

**Authors:** Mariana P. Proenca

**Affiliations:** 1 ISOM and Departamento de Electrónica Física, Universidad Politécnica de Madrid, Avda. Complutense 30, 28040 Madrid, Spain; mariana.proenca@upm.es; 2 IFIMUP and Departamento de Física e Astronomia, Faculdade de Ciências Universidade do Porto, Rua do Campo Alegre 687, 4169-007 Porto, Portugal

Low-dimensional magnetic nanostructures are promising materials for applications in many different fields, for example, in medicine (for drug delivery, biomimetic devices, contrast agents in magnetic resonance imaging, hyperthermia, cell manipulation, etc.), in informatics (for security systems, information storage devices, quantum computing, etc.), and in engineering (for magnetic sensors and actuators, energy harvesting, catalysis and magnetic separation, spintronic-based devices, etc.). Reducing materials to the nanoscale allows new properties to emerge. Their correct tuning will progress the design of new and exciting devices. This Special Issue summarizes some of the most recent works on the synthesis, characterization, theoretical modelling, and applications of 1D and 0D magnetic nanostructures.

This issue comprises a total of eight contributions: seven research papers [1,2,3,4,5,6,7] and one review article [8]. The research papers include: the design and modelling of racetrack memory devices using cylindrical nanowires (NWs) [1]; the study of the magneto-transport properties of interconnected arrays of magnetic NWs and nanotubes [2]; the synthesis, characterization and micromagnetic simulation of multi-segmented [3] and diameter-modulated [4,5] magnetic NW arrays; the implementation of new techniques to improve the large-scale fabrication of electrodeposited magnetic NW arrays [6]; and the synthesis and characterization of magnetic nanoparticles, aiming at their application as contrast agents in magnetic resonance imaging (MRI) [7]. Finally, the included review article [8] is focused on the influence of magnetic anisotropy, post-processing conditions, and defects on the domain wall dynamics in magnetic microwires. 

In the work of Rial et al. [1], a new design of a 3D racetrack memory device using multi-segmented and/or diameter-modulated cylindrical magnetic NWs is proposed. This is a theoretical work in which several NW configurations are simulated, aiming at the future integration of the writing heads along the wire, thus avoiding the need to implement external writing heads next to the track. These integrating writing heads would be composed of a bilayered NW section with two different diameters, compositions, or crystallographic structures. The simulated results showed that the magnetic information could be written by applying external magnetic fields and then moved to the storage elements using spin-polarized currents through the wires.

The works of [2,3,4,5] then show experimental evidence of the influence of several geometrical parameters on the magnetic properties of NW arrays.

Da Câmara Santa Clara Gomes et al. [2] measured the magneto-transport in 3D networks of interconnected magnetic NWs and nanotubes. Their results illustrate the strong influence of the diameter, packing density, angle distribution and tube wall thickness on the magnetic and magneto-transport properties of the interconnected networks. The authors also found giant magnetoresistance responses when adding non-magnetic layers intercalated with the ferromagnetic ones in interconnected multi-layered NW networks.

Caspani et al. [3] studied the effect of the number of magnetic layers and their thickness on the magnetic properties of multi-segmented ferromagnetic/non-magnetic NW arrays. Their results showed that when the ferromagnetic layers were thinned to 20–30 nm and separated by more than 60 nm along the wire length, they behaved like isolated ferromagnetic disks. Moreover, in the particular case of Fe/Cu bilayered NWs with 20 nm of the Fe segments’ length, a synthetic antiferromagnetic system was formed, in which the coercivity and remanence values were close to zero.

The works of García et al. [4] and Arzuza et al. [5] focused on the magnetic properties of diameter-modulated cylindrical NWs. García et al. [4] studied the effect of a sharp transition between the two different diameters in the magnetization reversal process of the bi-segmented NWs. After analysing both the NW arrays and individual NWs, the authors found that the magnetization reversal was carried out in two steps. Micromagnetic modelling illustrated a core–shell reversal mechanism, in which the shell of the wider segment reversed first, followed by its core together with the narrower segment.

In the work of Arzuza et al. [5], the bi-segmented NWs presented a smoother transition between the different diameters. The main aim of this work was to understand the effect of the length/diameter aspect ratio of the wider segment in the magnetization reversal of the bi-segmented NW arrays. Using the first-order reversal curve (FORC) method combined with micromagnetic simulations, the authors found that wide segments with lower aspect ratios promoted the stabilization of pinned domain walls at the diameter modulation during magnetization reversal.

Finally, Fernández-González et al. [6] developed a new route to fabricate large quantities of magnetic NWs with reduced costs and production time. The authors proposed several modifications to the conventional electrochemical growth of NWs, in particular, the use of soft anodization processes of recycled aluminium at room temperature to produce the nanoporous alumina templates and galvanostatic deposition methods to grow CoFe NWs inside the pores. Their results showed that the morphology, composition, and magnetic properties of the produced NWs were very similar to those of NWs obtained by conventional methods.

Apart from the 1D magnetic nanostructure (NWs and nanotubes), this Special Issue also includes one research article dedicated to 0D nanostructures, namely iron oxide nanoparticles (IONPs), as MRI contrast agents. The work of Cortés-Llanos et al. [7] shows how the IONPs’ core size and the use of distinct coatings may affect their aggregation and interactions in different media, as well as their cell labelling efficiency and in vitro MRI. Their results demonstrated that the greatest labelling occurred when using IONPS with hydrodynamic sizes greater than 300 nm. As for MRI applications, the best contrast was obtained when using IONPs with core sizes between 12–14 nm coated with dimercaptosuccinic acid.

To conclude, Zhukova et al. [8] published a review article on the influence of several fabrication techniques on the domain wall (DW) dynamics of magnetic microwires. For example, minimizing the magnetoelastic anisotropy, by tuning the chemical composition of the microwires or performing heat treatments, would improve DW dynamics (velocity and mobility). In particular, stress annealing was found useful for the design of a magnetic anisotropy distribution in amorphous microwires that would promote an increase in DW velocity.

I hope the readers will find this Special Issue a good selection of works on multifunctional magnetic micro- and nanowires, nanotubes and nanoparticles.
